# Detection of Singlet Oxygen Formation inside Photoactive Biohybrid Composite Material

**DOI:** 10.3390/ma11010028

**Published:** 2017-12-26

**Authors:** Kata Hajdu, Ateeq Ur Rehman, Imre Vass, László Nagy

**Affiliations:** 1 Institute of Medical Physics and Informatics, University of Szeged, Rerrich B. sq. 1, 6720 Szeged, Hungary; L.Nagy@physx.u-szeged.hu; 2 Isotope Climatology and Environmental Research Centre (ICER), Institute for Nuclear Research, Hungarian Academy of Sciences, Bem tér 18/c, 4026 Debrecen, Hungary; 3 Biological Research Center of the Hungarian Academy of Sciences, Institute of Plant Biology, Temesvári krt 62, 6726 Szeged, Hungary; ateeq@brc.hu (A.U.R.); vass.imre@brc.mta.hu (I.V.)

**Keywords:** photoactive nanocomposites, singlet oxygen, photosynthetic reaction center, multiwalled carbon nanotubes

## Abstract

Photosynthetic reaction center proteins (RCs) are the most efficient light energy converter systems in nature. The first steps of the primary charge separation in photosynthesis take place in these proteins. Due to their unique properties, combining RCs with nano-structures promising applications can be predicted in optoelectronic systems. In the present work RCs purified from *Rhodobacter sphaeroides* purple bacteria were immobilized on multiwalled carbon nanotubes (CNTs). Carboxyl—and amine-functionalised CNTs were used, so different binding procedures, physical sorption and chemical sorption as well, could be applied as immobilization techniques. Light-induced singlet oxygen production was measured in the prepared photoactive biocomposites in water-based suspension by histidine mediated chemical trapping. Carbon nanotubes were applied under different conditions in order to understand their role in the equilibration of singlet oxygen concentration in the suspension. CNTs acted as effective quenchers of ^1^O_2_ either by physical (resonance) energy transfer or by chemical (oxidation) reaction and their efficiency showed dependence on the diffusion distance of ^1^O_2_.

## 1. Introduction

The fabrication of photoactive bio-nanocomposites bears high interest because of their wide range of versatile application possibilities. In spite of this attention, the function of these systems still needs further investigation to enhance their efficiency. For this, it is essential to understand the functioning of the natural light harvesting systems that own inimitable punctuality and precision during the harvesting of light. Different biological systems are used [1,2,3,4,5,6,7,8] to create new generation light harvesting systems. In these composites the advantages of the biological components and the carrier matrices are combined so that these systems can be state of the art, really smart, modern and innovative devices.

The first steps of the primary charge separation in photosynthesis take place in the photosynthetic reaction centres (RCs). These pigment−protein complexes are integrated in the photosynthetic membranes of plant and bacterial cells and they are able to harness the energy of every absorbed photon with quantum efficiency near to 100%. Although it is developed in nanometer scale, and is working in “nanoscopic work” (it converts the energy of a single photon), this protein assures the energy input practically for the whole biosphere. The capture of light energy by chlorophylls (or bacteriochlorophylls in photosynthetic bacteria, which is the subject of our research) results in the separation of positive and negative charges, so P^+^BPheo^−^ state is followed by the P^+^Q_A_^−^ state, where P^+^ is the oxidized primary electron donor, a specialized bacteriochlorophyll dimer, (BChl)_2_, BPheo is the first electron acceptor, a monomer bacteriopheophytine, and Q_A_^−^ is the reduced quinone-type primary electron acceptor. The separated charges are then further stabilized in the form of the P^+^Q_B_^−^ redox state, where Q_B_^−^ is the reduced secondary quinone [9,10,11]. Besides the fact that there is a solid knowledge about RC isolated from *Rhodobacter sphaeroides*, it is easy to engineer (either by genetic engineering or by protein biochemistry after the preparation) and to prepare, it contains only three protein subunits, and stable for long time. Because of its advantageous properties, there are several attempts to use RC for special integrated optoelectronic (e.g., light harvesting) applications [12,13,14,15].

It must be mentioned that besides the bacterial RCs, photosynthetic reaction enters isolated from cyanobacteria and plants, photosystem I (e.g., [16,17,18,19] and PS II [20,21,22,23] reviewed, e.g., by [24] are also used in photoelectrochemical cells, energy conversion or optoelectronic applications.

Earlier studies have proven that carbon nanotubes (CNTs) are suitable carriers for various biomolecules and electron transport can be observed between them [25,26,27,28,29]. This way, they can function not only as stabilizing, but also a functionally active matrices in optoelectronic applications, sensors, etc. [30,31]. The electrical and mechanical properties of carbon nanotubes and other carbon-based materials became the focus of several research projects in the last twenty-five years [32]. Depending on the application, different functionalisation techniques are widespread on single- and multiwalled carbon nanotube surfaces [33,34]. The process is well controllable that results in different functional groups on the surface (like -COOH, -NH_2_), making the CNTs appropriate to be integrated in hybrid materials. Combining RC with single- or multiwalled CNTs with the use of different binding techniques, a photo-active hybrid material is created where the efficient light energy harness of RC is coupled with the good conductivity of CNT. Electron transport was detected between the two materials. CNTs do not modify the direct electron transport between the redox cofactors of the RC but stabilise the charge separation so the life time of the separated charge pair is extended [19]. The long-term activity of the RC/CNT depends on many properties, internal and external factors as well.

After photoexcitation, light energy is converted into chemical potential with near to 100% efficiency, meaning that every absorbed photon initiates separation of a single charge pair inside the RC. During this process triplet states of the redox active components of the electron transfer can be formed with a certain probability, which leads to the formation of singlet oxygen and other reactive oxygen species (ROS). Singlet oxygen (^1^O_2_) is a highly reactive molecular form of oxygen that is produced—besides other reactive oxygen species (ROS)—during many photochemical and photobiological processes. The arising singlet oxygen can react chemically with the present biomaterial and can destroy its structure after photoexcitation [35,36,37,38,39,40]. These aggressive chemical reactions are able to shorten the life time of biocomposites due to their photodegradation. In order to take advantages of the effectiveness of these unique materials, it is important and a real challenge to find the most appropriate conditions and keep their photoactivity for long period.

During photosynthetic processes, singlet oxygen is generated usually in fluidic phase by a photosensitizer. When the excited triplet state of the sensitizer reacts with oxygen, it relaxes back to the ground state and ^1^O_2_ is formed as a result of the reaction. The diffusion distance of ^1^O_2_ is really small because of the short life time (0.05–25 µs, depending on the place of formation and physiological factors) [41,42,43,44,45]. There are routine techniques for detection of ^1^O_2_, like measuring light absorption or fluorescence of specific dyes, spin trapping EPR probes, etc., which can also be used in biological samples with special attention to the system [46,47,48,49,50]. A simple but efficient method was published by Telfer et al. in 1994 [51]. These authors used histidine to capture singlet oxygen with its imidazole rings, forming a dioxygen complex, so the decrease of bulk oxygen concentration can be monitored precisely by an oxygen electrode.

Thanks to their extended π- conjugated orbital system carbon nanotubes (CNTs) are also known to be able to produce ^1^O_2_ [52], however—under certain conditions—they can also act as efficient quenchers, depending on the external factors [53,54,55,56,57]. The way of the reaction between the carbon nanotubes and reactive oxygen species (ROS) depends not only on the surrounding environment, but also on the type, the purity and the functionalization of the carbon nanotubes [58]. The aim of the project was to characterize the effect of carbon nanotubes on singlet oxygen formation in bio-nanocomposites formed by bacterial RCs and CNTs after light excitation, and understand the ongoing light-induced reactions, which is highly affected by the complexity and stability of this hybrid system. Carbon nanotubes are seemed to be effective quenchers of the singlet oxygen formed after the photoexcitation of this bio-nanohybrid system.

## 2. Results and Discussion

**Figure 1:**
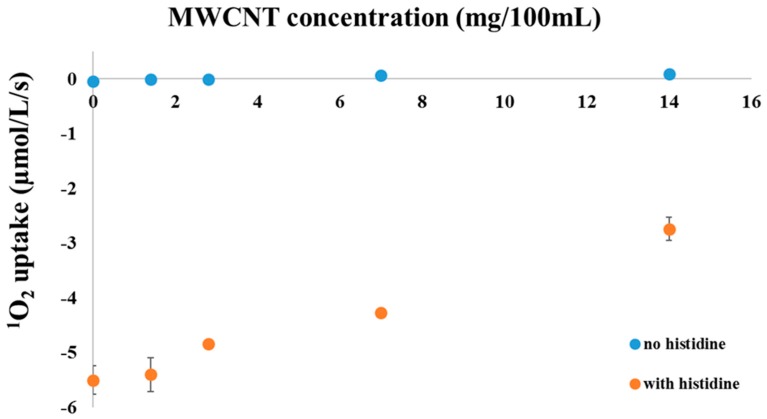
Oxygen uptake of 1 µM methylene blue solution in the presence (orange circles) or absence (blue circles) of 5 mM histidine, measured with a Clark type electrode as a function of the concentration of the carboxyl functionalized multiwalled carbon nanotubes.

Clark type O_2_ electrode was used to measure the rate of singlet oxygen by chemical trapping. As a result of the reaction between the imidazole ring of histidine and the produced singlet oxygen, the amount of the total dissolved oxygen decreases in the suspension [59,60]. The rate of His-mediated O_2_ uptake is equal to the rate of ^1^O_2_ formation provided that the added His is in sufficient surplus to react with all ^1^O_2_ molecules. According to previous data 5 mM His is sufficient to saturate the O_2_ uptake in photosynthetic systems, i.e., eliminate all produced ^1^O_2_. Application of this method demonstrated light induced ^1^O_2_ production by 1 µM methylene blue (MB), which is used as a standard sensitizer of ^1^O_2_ production complexes (Figure 1). In order to see the effect of MWCNTs on ^1^O_2_ formation, the rate of MB induced ^1^O_2_ formation was also checked in the presence of different concentrations of MWCNTs.

Thanks to their extended π-conjugated orbital system, carbon nanotubes are theoretically capable to interact with oxygen molecules both in their triplet ground state and singlet excited state, and the literature data indicate that they can act both as ^1^O_2_ generators and quenchers [52,53,54]. Under our experimental conditions (visible and near infrared light illumination, room temperature is controlled in water bath and the sample was stirred with magnet bar) we did not find oxygen uptake with MWCNT-COOH alone (data are not shown). Consequently, we checked if the MWCNT-COOH nanotubes can act as ^1^O_2_ scavengers, or not. To this end we performed an experiment to check the efficiency of His-mediated trapping of ^1^O_2_, which was generated by illumination of methylene blue (1 µM, phosphate buffer pH 7.0), influenced—presumably—by the presence of different concentrations of MWCNT-COOH (in suspension).

In the absence of histidine no considerable oxygen uptake, i.e., no ^1^O_2_ production, was detected within the sensitivity of the O_2_ electrode regardless of the presence or absence of MWCNT-COOH. After the addition of 5 mM histidine in the absence of any MWCNT-COOH a significant oxygen uptake was measured due to His-mediated chemical trapping of ^1^O_2_ which was produced by illumination of methylene blue. In the presence of added MWCNT-COOH the rate of O_2_ uptake decreased in a concentration dependent manner, which shows that the concentration of ^1^O_2_, which is available for interaction with His is decreased. This finding demonstrates that MWCNT-COOH nanotubes possess a ^1^O_2_ scavenging ability and compete with His to interact with ^1^O_2_. The most likely explanation of the results is that MWCNT-COOH nanotubes quench ^1^O_2_ predominantly via a physical mechanism, i.e., via energy transfer from ^1^O_2_ to the nanotubes, which converts the excited ^1^O_2_ back to ground state O_2_ without eliminating dissolved O_2_ molecules.

**Figure 2:**
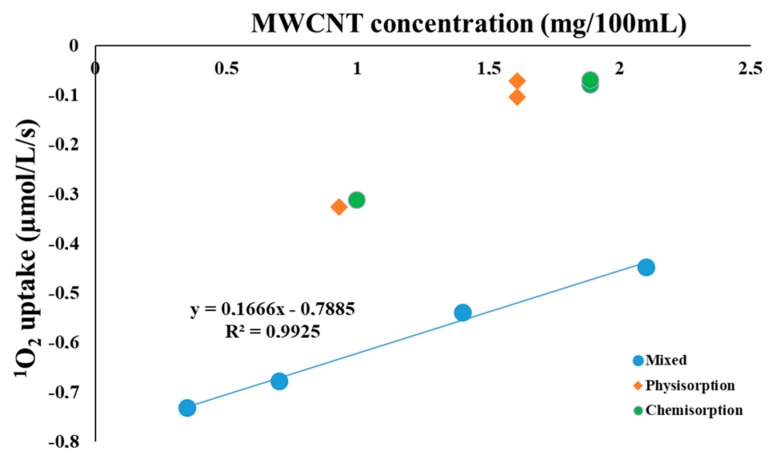
Oxygen uptake of the RC solutions prepared by different procedures and measured with a Clark type oxygen electrode as a function of the MWCNT concentration. The reaction mixture contained 1 µM RC and 5 mM histidine. RCs were bound to MWCNT-COOH with different concentrations and binding methods, or were just mixed with the MWCNTs as indicated.

After examining the effect of MWCNT on the ^1^O_2_ concentration in the model system in which methylene blue was used as a sensitizer, we were interested in the characteristics of the ^1^O_2_ generation by the RC/MWCNT-COOH system. In this composite, reaction center purified from *Rb. sphaeroides* R-26, a carotenoid-less strain of purple bacteria, acts as ^1^O_2_ sensitizer, and MWCNT-COOH might act as a quencher either by physical or by chemical mechanisms. First, 1 µM RC was mixed with MWCNT-COOH in different concentrations. Second, RC was bound to MWCNT-COOH by physical sorption and third, by EDC/NHS chemical crosslinking methodology. In both cases, sorption was done in two different RC-MWCNT ratios. Light induced ^1^O_2_ uptake was measured for all the three suspensions (Figure 2).

The data show that MWCNT-COOH has a significantly increased ^1^O_2_ quenching efficiency when RC complexes are in physical contact or direct chemical interaction with the nanotubes. Singlet oxygen is generated by the bacteriochlorophyll triplets accompanying the photochemistry inside the RC. Possible roles of the MWCNT in the deactivation of the excited bacteriochlorophylls—either direct energy transfer between the chromophores and carbon nanotubes or through formation of ^1^O_2_ from its ground state [61]—are already proposed, however, mechanisms are not clarified yet. When RC is bound to the MWCNT, either through physical or chemical binding, due to the close contact both mechanisms can be accounted in a competitive manner, which reduces the yield of the measured ^1^O_2_. In the case of close contact, the distance between RC and MWCNT is well within the diffusion path of the ^1^O_2_, which is estimated to be 90–120 nm [45]. As it was expected, when RC and MWCNTs are only mixed in the suspension without sorption procedure, the oxygen uptake, reflecting ^1^O_2_ concentration which is available for chemical trapping, is larger and shows a concentration dependence, indicating that the ^1^O_2_ quenching process is limited mainly by diffusion. Further proof of this idea is that extrapolation of the measured oxygen uptake values to the MWCNT free samples results in almost the same values for both composite samples (0.7–0.8 µM^1^O_2_/L/s, within a possible experimental error).

**Figure 3:**
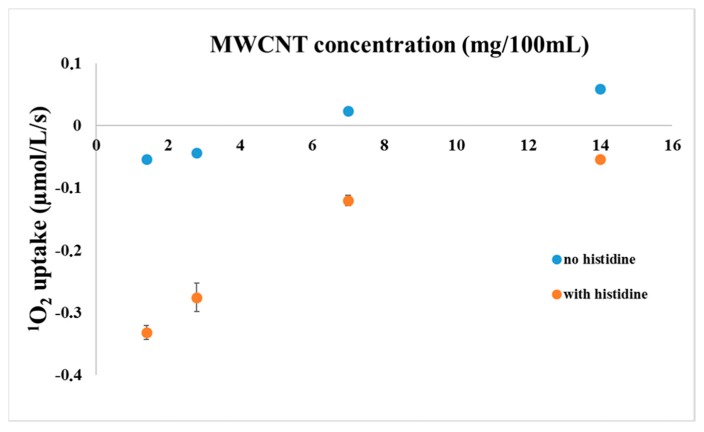
Oxygen uptake of mixed solutions of RC and MWCNT-COOH measured with a Clark type electrode as a function of MWCNT concentration in the presence or absence of histidine, as indicated. The reaction mixture contained 1 µM RC and 5 mM histidine (if added).

As a control experiment, oxygen uptake of mixed solutions of RC and MWCNT-COOH were measured with the Clark electrode as a function of MWCNT concentration in the presence or absence of histidine (Figure 3). As it was expected, due to the sensitization by the RC in the presence of histidine, an oxygen uptake was measured in a concentration dependent manner. However, in the absence of histidine, the yield of trapped ^1^O_2_ was reduced considerably. The oxygen uptake measured in the absence of histidine is probably due to the chemical reaction of ^1^O_2_ with MWCNT-COOH, so chemical quenching is one of the possible reaction mechanisms between these two materials. Note, that ^1^O_2_ interacts in the same way with the Clark electrode as normal O_2_, i.e., ^1^O_2_ production in itself does not change the level of dissolved O_2_.

Interestingly, above a certain concentration of the MWCNT-COOH, the change in the oxygen concentration became positive indicating a generation of oxygen in the reaction chamber rather than an uptake. This finding can be explained if MWCNT acts not only as a quencher of the ^1^O_2_ but a source of O_2_ as well. The actual increase of dissolved O_2_ level can be explained if functional groups found on MWCNTs after functionalization are split to O_2_ after light induced singlet oxygen formation by the RC. The best candidates to these groups are peroxides or superoxides, the presence of which should be verified.

There are indications that CNT may act as ^1^O_2_ sensitizer under prolonged UV irradiation [52]. However, this effect is not very probable under our experimental conditions because of using white light tungsten ball illumination, which is mainly rich in IR range of the spectrum [46], and occurs through the UV absorbing plastic wall of the measuring chamber.

**Figure 4:**
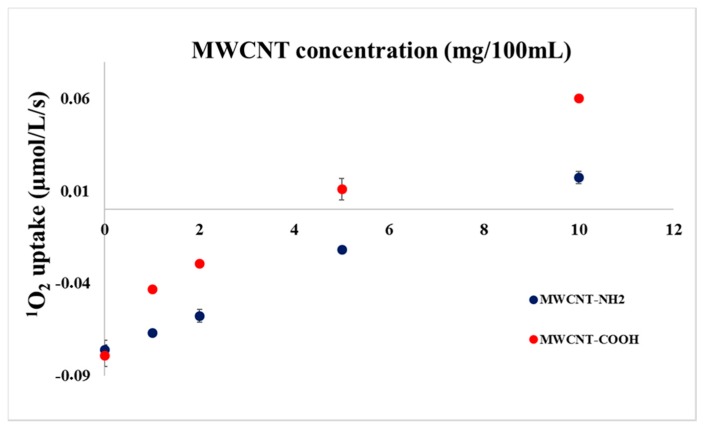
Oxygen uptake of mixed solutions of RC and MWCNT-COOH or MWCNT-NH_2_ measured with a Clark type electrode as a function of MWCNT concentration in the presence of 1 µM RC.

It was already proved [46,61,62] and our experiments also indicate that MWCNT functionalization plays important role when deactivation of singlet oxygen is concerned. In order to test the effect of the functional groups in our experimental arrangement, experiments were carried out in the presence of 1 µM RC but in the absence of histidine, with MWCNTs functionalized with amine (MWCNT-NH_2_) or carboxyl (MWCNT-COOH) groups. Results are summarized in Figure 4.

The reaction of carbon nanotubes with singlet oxygen is a complex mechanism. Without MWCNT and histidine only a small oxygen uptake (ca. 0.08 µM^1^O_2_/L/s) was measured in both experiments due to the presence of reactive functional groups. The effect decreases gradually when MWCNT is added either functionalized with carboxyl—or with amine groups. The deactivation seems to be more pronounced when carboxyl-functionalized MWCNT is added. This result is in line with the results of Boldog et al. [46] who also found enhanced deactivation with MWCNT-COOH as compared with MWCNT-NH_2_. However, these authors used specific dye, DPBF, for the detection of singlet oxygen. Positive turn in the oxygen concentration change was found for both samples, however, at a larger concentration for MWCNT-COOH. This result indicates that because of the highly oxidising environment during the preparation of MWCNT-COOH, considerable amount of oxygenic chemical groups is situated on the MWCNT-COOH surface. These oxygenic chemical groups can facilitate the release of oxygen after contacting reactive singlet oxygen an in this case, positive parallel shifts can be assumed, caused by the O_2_ elimination from MWCNTs.

## 3. Materials and Methods

### 3.1. Sample Preparation

Photosynthetic reaction center protein was purified from *Rhodobacter (Rb.) sphaeroides* R-26, a mutant strain of purple bacteria, which lacks carotenoids (derived from the laboratory of Colin Wright, University of Illinois, Urbana, IL, USA). Cells were grown photo-heterotrophically under anaerobic conditions in a Siström-medium supplemented with potassium succinate [63]. RCs were prepared by LDAO (lauryldimethilamine N-oxide, Fluka Chemie Ag., Buchs Switzerland) solubilization and standard protein purification methods (ammonium sulfate precipitation, DEAE Sephacell (Sigma, St. Louis, MO, USA) column chromatography and ultrafiltration) as described previously [64]. The purified RCs were bound to carboxyl-functionalised multi-walled carbon nanotubes (MWCNT) through physical sorption or through EDC/NHS chemistry procedure [61]. MWCNT was activated by the addition of crosslinkers N-hydroxysuccinimide (NHS) and 1-[3-dimethylaminopropyl]-3-ethyl-carbodiimide (EDC). After activation, the mixture was dialyzed in potassium phosphate buffer (0.1 M, pH 7.0) then, the ca. 100 M RC was added to the activated MWCNT and it was stirred at 4 °C for 3 h. Finally, the sample was separated and washed by an ultracentrifuge until the steady-state absorption spectrum of the supernatant did not show the characteristic peaks of the RC. The amount of the immobilised RC was calculated by spectroscopic measurements (UNICAM UV-4 double-beam spectrophotometer) by using an extinction coefficient of ***ε***_802 nm_ = 288 mM^−1^ cm^−1^ [65].

MWCNTs were synthesized in the laboratory of Professor Klara Hernadi (University of Szeged, Szeged, Hungary) by catalytic chemical vapor deposition (CCVD) in the presence of acetylene as carbon source. Functionalization of MWCNTs with carboxyl groups was carried out in the same laboratory in aqueous nitric acid solution with a concentration of 10 m/m% for 1 h.

### 3.2. Singlet Oxygen Production Measurements

Singlet oxygen concentration was determined by His-mediated chemical trapping measuring oxygen uptake with a Hansatech DW2/2 Clark type electrode in liquid phase (1 µM RC; 5 mM histidine, phosphate buffer pH 7.0, 30 °C, light intensity 500 µE) as described earlier [60]. The rate of O_2_ uptake was used as a measure of ^1^O_2_ production. The extent of ^1^O_2_ production was expected to be proportional to the amount of O_2_ uptake from the aqueous solution [66]. The reactions of ^1^O_2_ with Histidine are very specific. Initially this reaction generates the production of short lived endoperoxides, followed by the production of stable oxidation products, which chemically trap singlet oxygen in aqueous solution [67]. The effect of carbon nanotubes was detected in mixed samples, and also after immobilizing the RC on the MWCNT surface with physical sorption or chemical binding, as described above.

## 4. Conclusions

In this study singlet oxygen production was measured by using a Clark type electrode after light excitation of photosynthetic reaction centre (RC) purified from purple bacteria *Rb. sphaeroides* R-26. Carotenoid less RCs produce singlet oxygen with high yield accompanying photoreaction. The main goal of the study was to characterise the effect of carbon nanotubes on the ^1^O_2_ production in RC/MWCNT photoactive hybrid materials. Clarke type oxygen electrode was well applicable and efficient tool to measure ^1^O_2_ concentration in the biohybrid suspension. Different RC/MWCNT composites were tested. RC was either mixed or bound (by physical or chemical sorption) with carboxyl functionalised MWCNT. In all these cases, higher MWCNT concentrations resulted in less ^1^O_2_ in the suspension. Carbon nanotubes reacted with and quenched the produced, highly reactive singlet oxygen. It was proven that the distance between the RCs and MWCNTs is a determining factor of quenching efficiency, probably due to the diffusion path. The biggest quenching effect was visible after physical sorption, where RC is bound directly on the MWCNT surface, without any crosslinker bridges. In the case of mixed samples, the quenching effect was smaller due to the larger distance between the interacting components and also because of the micelle system which is necessary to keep the system stable in the water phase.

^1^O_2_ quenching proceeds in physical and chemical ways, and both of these mechanisms has to be considered in case of carbon nanotubes. We made an attempt to clarify which mechanism is preferable in the RC/MWCNT systems, so that measurements were done with and without chemical trapping of ^1^O_2_ by histidine. Measurements showed considerable difference—in the absence of histidine smaller change was detected compared to the equilibration oxygen concentration. In addition, positive turn in the oxygen concentration change was recorded. CNTs acted as quenchers—and not synthesizers—of ^1^O_2_, that can be one reason for the increased stability and efficiency of photosynthetic systems when attached to them. It is reasonable to assume, that MWCNT quenched the ^1^O_2_ preferentially through physical interaction under our experimental conditions however chemical quenching is also perceptible. According to our rough estimations based on mass concentration ratios (note that molar concentration cannot be applied in case of CNTs), approximately two order of magnitude higher chemical quenching efficiency values can be calculated for MWCNTs as compared to histidine.

Beyond the quenching another effect was found for both types of functionalized MWCNT (MWCNT-COOH and MWCNT-NH_2_) samples. The explanation of the positive change in the O_2_ concentration is the increase of oxygen content in the electrode environment. The possible sources of the “extra O_2_” are functional groups on the surface of the MWCNT, mostly in case of MWCNT-COOH where the functionalisation procedure requires a highly oxidative environment. These oxygenic groups can split to O_2_ upon light excitation, and synproportionation with the uprising ^1^O_2_ has to be also considered. Further investigation about the possible source of oxygen is under progress in our laboratory.
